# Delayed-onset of progressive pseudorheumatoid dysplasia in a Chinese adult with a novel compound *WISP3* mutation: a case report

**DOI:** 10.1186/s12881-017-0507-3

**Published:** 2017-12-15

**Authors:** Qiongyi Hu, Jing Liu, Yi Wang, Jiucun Wang, Hui Shi, Yue Sun, Xinyao Wu, Chengde Yang, Jialin Teng

**Affiliations:** 1 Department of Rheumatology and Immunology, Ruijin Hospital, Shanghai Jiao Tong University School of Medicine, Shanghai, 200025 China; 2 State Key Laboratory of Genetic Engineering and Ministry of Education (MOE) Key Laboratory of Contemporary Anthropology, Collaborative Innovation Center for Genetics and Development, School of Life Sciences, Fudan University, Shanghai, China

**Keywords:** Progressive pseudorheumatoid dysplasia-PPD-WISP3-skeletal dysplasia-mutation

## Abstract

**Background:**

Progressive pseudorheumatoid dysplasia (PPD) is a rare autosomal recessive genetic disease that is characterized by pain, stiffness and enlargement of multiple joints with an age of onset between 3 and 8 years old. Mutations in the *WISP3* (Wnt1-inducible signal pathway) gene are known to be the cause of PPD.

**Case presentation:**

We present a case of delayed-onset PPD in a Chinese man. The 35-year-old proband presented with an almost 20-year history of pain and limitations in mobility in multiple joints. Based on the clinical manifestations, the patient was diagnosed with PPD; however, there was no specific evidence to confirm this diagnosis. Through mutational analyses, two *WIPS3* mutations in exon 4, including a novel frameshift mutation (c.670dupA) in the paternal allele and an already described nonsense mutation (c.756C > A, p.Cys252*) in the maternal allele, were identified in the proband. Thus, the patient was diagnosed with PPD. Furthermore, we found that the proband’s son only carried one of the mutations (c.670dupA) and therefore determined that he would not be affected by PPD in the future.

**Conclusions:**

In this case, we successfully diagnosed the disease that the proband was affected precisely after the reunion of clinical diagnosis and genetic analysis. These findings demonstrate the clinical utility of genetic analysis to diagnose skeletal dysplasia and guide genetic counseling.

## Background

Progressive pseudorheumatoid dysplasia (PPD; MIM208230) is an extremely rare and inheritable autosomal recessive disease with an approximate incidence of one per million in the UK [1]. This disorder is characterized by non-inflammatory arthropathy. The signs and symptoms of the disorder typically begin between the ages of 3 and 8, and the typical manifestations include pain, stiffness and limitation in motion of multiple joints in the absence of extra-skeletal involvement and bone erosion [2]. Patients with atypical presentations manifest as seronegative arthropathy either under 3 years old or above 8 years old [3].

PPD has been attributed to the homozygous or compound heterozygous loss-of-function mutations in *WISP3* (Wnt1-inducible signal pathway; MIM: 603,400), which is located on chromosome 6q22 [4]. WISP3 is a member of the connective tissue growth factor (CCN) family of cysteine-rich growth factors [5]. It has been reported that WISP3 influences cartilage homeostasis and bone growth [4]. To date, more than 64 different *WISP3* mutations have been reported in more than 200 PPD individuals [6], with only 18 from Chinese patients.

Here, we report a case of delayed-onset PPD in a 35-year old Chinese man. Using genetic analysis, we identified a novel compound *WISP3* mutation in exon 4, a frameshift mutation (c.670dupA) in the paternal allele and a nonsense mutation (c.756C > A, p.Cys252*) in the maternal allele in the proband. Additionally, we found that the proband’s son only carried one mutation (c.670dupA).

## Case presentation

The proband, a 35-year old man, was referred to us for a 20-year history of pain and limitation in mobility of multiple joints and a one-year history of worsening symptoms. He developed kyphosis at the age of 11. The patient endorsed pain in his palms, wrists and elbow joints without the presence of a rash or erythematous swelling. Subsequently, he progressed to develop pain and restricted movements in his hip and ankle joints that was associated with limited motion with squatting. A suspected diagnosis of congenital spondyloepiphyseal dysplasia was made at another hospital, and the doctor recommended he follow up without any therapy. The patient noted pain in his hips and knee joints, and he had difficulty in standing and sitting. The patient developed claudication and was only treated with occasional painkillers instead of systemic therapy.

At the age of 34, the patient was unable to walk without a crutch due to the pain and severely restricted motion in his hip joints after becoming very tired. At a local hospital, a laboratory test showed a normal erythrocyte sedimentation rate (2 mm/h), and tests for HLA-B27 remained negative. A radiograph of his hips showed sacroiliitis. The patient was suspected to have ankylosing spondylitis, and he was referred to our institution for further evaluation.

Examination of the spine showed an abnormal spinal curvature with limited range of motion in the cervical spine. Flexion contractures of the elbows and knees were observed, and there was limited range of motion in the elbows, shoulders, knees and ankles. The Patrick test and occiput-to-wall distance test were positive. The rest of his physical examination was unremarkable.

**Fig. 1:**
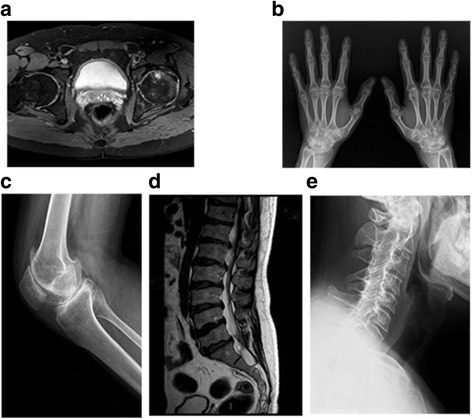
Radiographs of the proband at the age of 35 years. **a** Reduced interarticular spaces in the hip joints with bilateral femoral neck aseptic necrosis, **b** enlargement of interphalangeal joints, **c** narrowing of elbow joint space, **d** osteosclerosis and an irregular contour of the joint surface and narrow spaces in the knee joints, **e** herniated disks in the thoracolumbar spine (T12/L1-L5/S1) with lumbar spine hyperplasia, (**f**) and flattened vertebral bodies in the cervical spine can be noted

Radiological findings revealed the following: a reduced interarticular space of the hip joints with bilateral femoral neck aseptic necrosis (Fig. 1a); enlargement of the interphalangeal joints (Fig. 1b); osteosclerosis and irregular contour of the joint surface and narrowing of the spaces in the knee joints (Fig. 1c); herniated disks in the thoracolumbar spine with lumbar spine hyperplasia (Fig. 1d) and platyspondyly of the cervical spine (Fig. 1e).

**Fig. 2:**
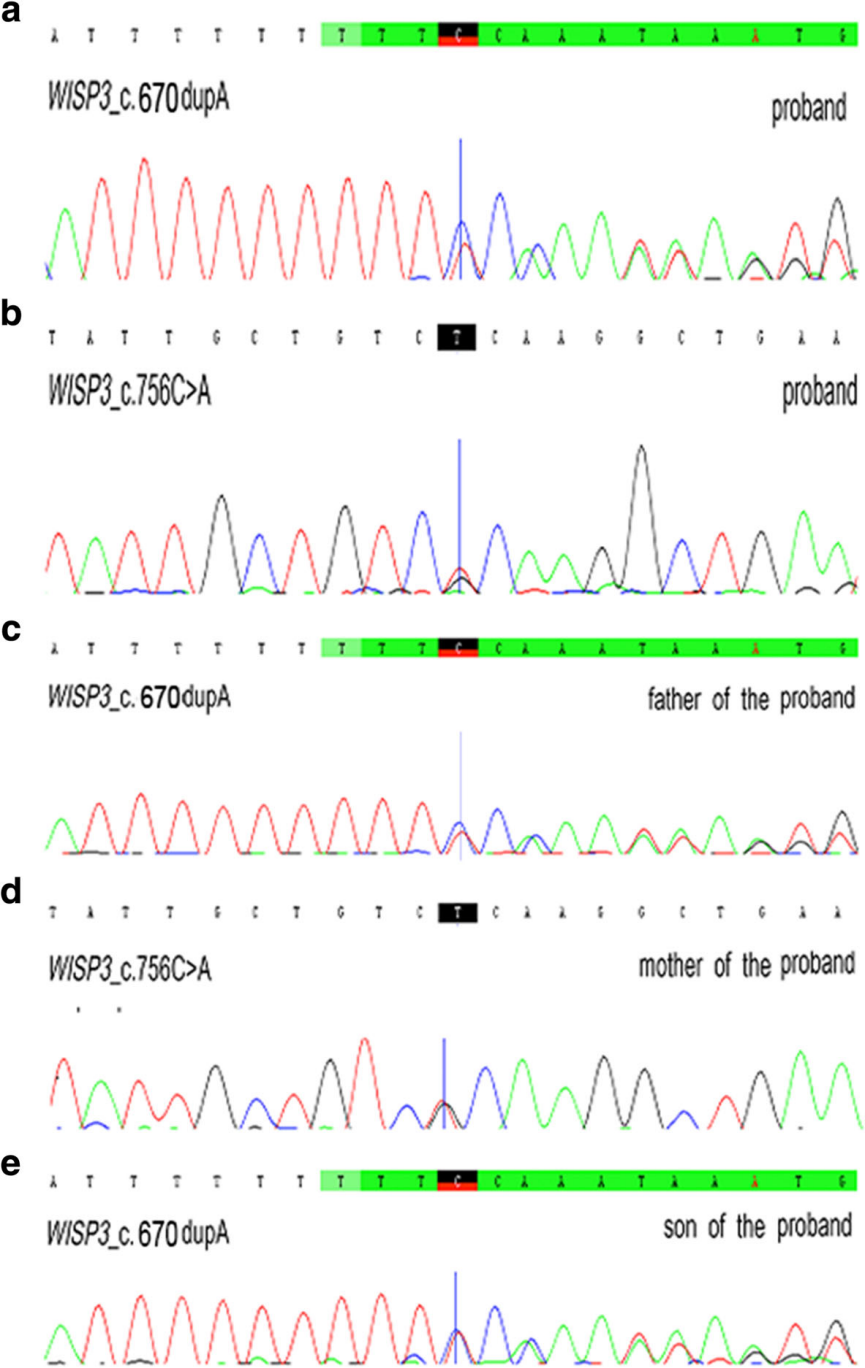
Sanger sequencing of *WISP3* mutations in the five family members. **a**, **b** Compound heterozygous mutations, including c.670dupA and c.756C > A, were found in the proband. **c** The c.670dupA mutation was inherited from the father. **d** The c.756C > A mutation was inherited from the mother. **e** The son carried the c.670dupA mutation

The rest of his five family members including his parents, wife and son did not have any evidence of skeletal dysplasia. Subsequently, the diagnosis of ankylosing spondylitis was challenged, and PPD was strongly suspected. A genetic analysis was carried out, following the CARE guidlines. We found that the proband carried a frameshift mutation (c.670dupA) and a nonsense mutation (c.756C > A, p.Cys252*) in exon 4 of *WISP3* (Fig. 2a, b), which have been reported previously [7, 8]. Furthermore, the frameshift mutation (c.670dupA) was found in the paternal allele (Fig. 2c), and the nonsense mutation (c.756C > A, p.Cys252*) was found in the maternal allele (Fig. 2d). The son of the proband was found to carry the frameshift mutation (c.670dupA) inherited from his grandfather (Fig. 2e).

A diagnosis of PPD was confirmed in the proband after combining the clinical characteristics and the results from the genetic analysis. Furthermore, genetic counseling was given for the son, who was told that he would not be affected by PPD in the future.

## Discussion and conclusions

PPD is a rare and inheritable skeletal dysplasia. In this report, we describe a 35-year old proband who suffered from non-inflammatory pain, stiffness and limitation in his mobility of multiple joints for over 20 years without a definite diagnosis. The proband had a normal blood laboratory test. Radiographs showed a narrowing of multiple joint spaces, enlargement of interphalangeal joints, herniated intervertebral disks, and platyspondyly.

Unlike other congenital skeletal dysplasias, patients with PPD are almost completely asymptomatic in early childhood [9]. The resemblance to juvenile idiopathic arthritis and lack of inflammatory biomarkers are the common causes of misdiagnosis. These diagnoses can be excluded by laboratory findings and genetic counseling [10]. Moreover, as patients younger than 3 years old or older than 8 years old can have atypical presentations of PPD [3], a diagnosis of PPD was initially considered. Genetic analysis was subsequently performed to confirm our original suspected diagnosis of PPD. To date, the incidence of PPD in China has not been described, and there is an urgent need to determine the spectrum of mutations in Chinese PPD patients and the relationship between the phenotype and genotype of PPD.

The causative role of *WISP3* mutations in PPD was first reported in 1999 [4]. Subsequently, a total of 18 *WISP3* mutations located in exons 2 to 5 have been described in China (Table 1) [2, 7, 8, 11–15]. They include five different mutations (two frameshift mutations and one nonsense mutation) located in exons 2 and 3 (two missense mutations), respectively, as well as five mutations in exon 5 (three frameshift mutations, one nonsense mutation and one missense mutation). To date, 8 *WISP3* gene mutations in exon 4 (c. 624delA, c.624_625insA, c.667 T > G, c.679dupA, c.716_722delAAATGAG, c.721 T > G, c.727_733delGAGAAAA, c.756C > A) have been identified in the Chinese population [7, 8, 13, 14].

**Table 1:** Summary of all currently known *WISP3* gene mutations in PPD patients in China

No.	Location	Nucleotide change	Amino acid change	References
1	Exon 2	c.105dupT	p.Gly36fs*10	[14]
2	Exon 2	c.208_209insA	/	[8]
3	Exon 2	c.136C > T	p.Gln46*	[2, 8, 13]
4	Exon 3	c.342 T > G	p.Cys114Trp	[8, 12–15]
5	Exon 3	c.342G > A	p.Cys114Try	[2]
6	Exon 4	c.624delA	p.Lys208fs*24	[14]
7	Exon 4	c.624_625insA	p.Cys209fs*229	[8]
8	Exon 4	c.667 T > G	p.Cys223Gly	[7, 8]
9	Exon 4	c.679dupA	p.Cys227Leufs*21	[15]
10	Exon 4	c.716_722delAAATGAG	p.Glu239fs*16	[12]
11	Exon 4	c.721 T > G	p.Cys241Gly	[15]
12	Exon 4	c.729_735delGAGAAAA	p.Glu243fs*255	[8, 13]
13	Exon 4	c.756C > A	p.Cys252*	[7]
14	Exon 5	c.840delT	p.Phe280Leufs*33	[11]
15	Exon 5	c.866_867insA	p.Gln289fs*31	[8, 12]
16	Exon 5	c.866dupA	p.Ser290Glufs*13	[13]
17	Exon 5	c.857C > G	p.Ser286*	[13]
18	Exon 5	c.1000 T > C	p.Ser334Pro	[11, 12]

In our study, we found compound heterozygous mutations (c.670dupA/ c.756C > A) in exon 4 of *WISP3* in an atypical PPD patient. These findings are different from the compound mutations in other Chinese patients with PPD previously reported by Ye et al. and Luo et al. [7, 8], indicating the genetic heterogeneity of PPD. Furthermore, to our knowledge, the c.756dupA *WISP3* mutation has not been previously reported in the Gene Bank database. In addition, the c.756C > A nonsense mutation was previously reported by Luo et al. in two patients from the same Chinese family [7]. In contrast, the phenotype in the patient in our study was milder than that of the patients described in their study, and the onset was much later. As typical PPD patients have an onset of symptoms between the ages of 3 and 8, the diagnosis of PPD in this patient was delayed for 20 years. Ye et al. described two delayed-onset PPD cases from the same family [8]. The age of onset was 13 and 14, respectively. Patients carrying a heterozygous mutation (c.208_209insA) show a significant difference in the severity of their PPD phenotype such as, coxa vara, genu varum, and loss of the joint space of the knee with osteoporosis. Due to the genetic heterogeneity of PPD, the correlation between the PPD phenotype and genotype requires further study.

WISP3 is a member of the connective tissue growth factor (CCN) family of cysteine-rich proteins that contains four functional modules. Module 3 is a thrombospondin repeated domain (TSP-1) encoded by exon 4 [5]. TSP-1, a 55-residue consensus sequence of the extracellular matrix glycoprotein, has the ability to bind to a wide range of extracellular targets, such as collagen V, fibronectin, sulphated glycoconjugates and several integrins, suggesting a role in adhesion or interactions with the extracellular matrix [16].

The WISP3 protein appears to have multiple effects on cartilage metabolism and homeostasis by regulating the synthesis of chondrocyte collagen type II and aggrecan, which are essential components in articular cartilage [17]. Zhou et al. found that WISP3 was expressed at low levels in articular cartilage chondrocytes in PPD patients [11], a finding that may explain why PPD mainly affects articular cartilage. However, the pathogenic mechanism of the *WISP3* gene in PPD remains unclear; thus, a specific therapy is not yet available [3]. Therefore, an early and definite diagnosis of PPD will be necessary to develop further studies.

In summary, a new combination of two *WISP3* mutations in exon 4 was identified in a Chinese adult with delayed-onset PPD. Genetic analysis led to a precise diagnosis of PPD. Our study demonstrated the clinical utility of genetic analysis to diagnose skeletal dysplasia and guide genetic counseling.

## Abbreviations

**CCN**: Connective tissue growth factor
**PPD**: Progressive pseudorheumatoid dysplasia
**TSP-1**: Thrombospondin repeated domain
**WIPS**: Wnt1-inducible signal pathway
